# Quantifying and Describing the Natural History and Costs of Alzheimer’s Disease and Effects of Hypothetical Interventions

**DOI:** 10.3233/JAD-191055

**Published:** 2020-06-02

**Authors:** Anders Wimo, Ron Handels, Bengt Winblad, Christopher M. Black, Gunilla Johansson, Stina Salomonsson, Maria Eriksdotter, Rezaul K. Khandker

**Affiliations:** a Department of Neurobiology, Karolinska Institutet, Care Sciences and Society, Division of Neurogeriatrics, Stockholm, Sweden; b Centre for Research and Development, Uppsala University/County Council of Gävleborg, Gävle, Sweden; c Department of Psychiatry and Neuropsychology, Maastricht University, Alzheimer Centre Limburg, School for Mental Health and Neurosciences, Maastricht, Netherlands; d Merck & Co., Inc., Center for Observational and Real World Evidence (CORE), Kenilworth, NJ, USA; e Merck Sharp and Dohme, Center for Observational and Real World Evidence (CORE), Stockholm, Sweden; f Department of Neurobiology, Karolinska Institutet, Care Sciences and Society, Division of Clinical Geriatrics, Stockholm, Sweden; g Theme Aging, Karolinska University Hospital, Huddinge, Sweden

**Keywords:** Alzheimer’s disease, cost analysis, cost effectiveness, costs, dementia, disease-modifying treatment, economics

## Abstract

**Background:**

A long-term horizon is necessary when the socioeconomic consequences and the potential effects of interventions in Alzheimer’s disease (AD) are estimated.

**Objectives:**

To illustrate the potential societal costs of AD across the disease continuum and to illustrate the potential cost-effectiveness of a hypothetical intervention with disease modifying treatment (DMT).

**Methods:**

Based on the Swedish dementia registry, a Markov model was used to simulate a virtual cohort of 100,000 people with mild cognitive impairment (MCI) due to AD (AD-MCI) in Sweden for 40 years starting at the age of 60. A simulated hypothetical intervention assumed a 25% reduction in progression rate during AD-MCI and mild AD-dementia. A comprehensive set of sensitivity analyses was included.

**Results:**

The cumulative risk to develop dementia was 96%. The mean simulated survival was 19.0 years. The net present value for a person year with dementia was 252,843 SEK (about 29,500 US$). The cost effectiveness model illustrated how the hypothetical scenario of a 25% reduction in progression to AD-dementia would require 41 AD-MCI patients to be treated to prevent one case of AD-dementia (2,447 avoided AD-dementia cases of 100,000 with AD-MCI). Most scenarios illustrated hypothetical cost effectiveness (based on a willingness to pay level of 600,000 SEK (70,000 US$) per gained QALY), but not cost savings.

**Discussion:**

Lifetime societal costs of AD are substantial. A future DMT may be potentially cost-effective given assumed treatment effects and costs, but cost savings are unlikely.

## INTRODUCTION

About 47 million people suffer from dementia worldwide [1] causing significant consequences for those with dementia and their families. The socioeconomic consequences are enormous: it was estimated that the global societal costs of dementia in 2015 were 817 billion US$ [2]. Future forecasts provide an even more challenging scenario with 75 million people affected in 2030 with societal costs of about 2 trillion US$. WHO stated in 2012 that dementia was a worldwide priority [3], resulting in the declaration at the World Health Assembly in May 2017 [4].

From epidemiological studies, several potentially modifiable risk factors for dementia have been identified [5]. More than 30% of dementia cases may be potentially preventable by modifying risk factors [6]. If the onset of dementia could be postponed by 2 years, the number of people with dementia in the USA could be reduced by 2 million in 50 years [7].

Most people with dementia suffer from Alzheimer’s disease (AD). Hitherto, only symptomatic drugs are available. Drugs under the label “disease-modifying treatment” (DMT) for AD have been the target for research, but so far, no such drug has entered the market, although there are several under development [5].

The period of cognitive impairment in dementia disorders such as AD, from the first signs to end of life state with dementia may last for 10–20 years or more [5]. By the introduction of imaging and biomarkers, it is also possible now to identify persons with an increased risk of developing AD. Thus, several international working groups have suggested a terminology that consider predementia states by expanding the AD concept to preclinical and prodromal states such as mild cognitive impairment (MCI) [8–14]. So far, this widening of the AD concept is mainly used for research purposes, but if DMTs become available, there will be a great need and challenge for considering predementia concepts in clinical practice [15, 16]. However, there is a large variation in the literature on estimates of the prevalence of MCI as well as the risk of progression to AD-dementia and other types of dementia [17–20].

From a prevention viewpoint, irrespective if the aim is lifestyle, risk factor management, or drug treatment or combinations, a program that starts in MCI due to AD (AD-MCI), and aims to influence the risk to progress to AD-dementia can be regarded as a secondary prevention treatment [21].

Resources are scarce and long-lasting programs to a wide population might have a large impact on care budgets. Thus, it is of vital interest to analyze not only the effectiveness of treatment (in terms of reduced morbidity and mortality) but also its cost-effectiveness.

The primary objectives were to estimate resource use, healthcare costs, and quality-adjusted life years of AD patients across the disease continuum as patient’s disease progresses. Secondary objectives were to illustrate the potential health-economic effects of a hypothetical AD-DMT using a decision-analytic model, with assumed treatment effect and costs.

### Ethics

The project was approved by the regional ethics committee in Stockholm (dnr 2016/2244-31).

## MATERIALS AND METHODS

### The SveDem registry

SveDem is a Swedish dementia registry, which started in 2007 and currently comprises over 90,000 people with different dementia disorders from the time of dementia diagnosis to annual follow-ups [22]. The registry was used to estimate the natural progression in dementia and AD. At the time of the study, we selected the data of 91,371 observations from 53,880 individuals with AD.

### Model overview

A Markov cohort model was constructed using a cohort of dementia patients with AD-MCI to simulate the societal health-economic burden of AD across the disease continuum. A hypothetical DMT intervention with assumed treatment effect and costs was evaluated using this model. To reflect the variability in the literature, a comprehensive set of sensitivity analyses was undertaken to test the uncertainty and robustness of the simulations.

### Basic model design

**Fig. 1:**
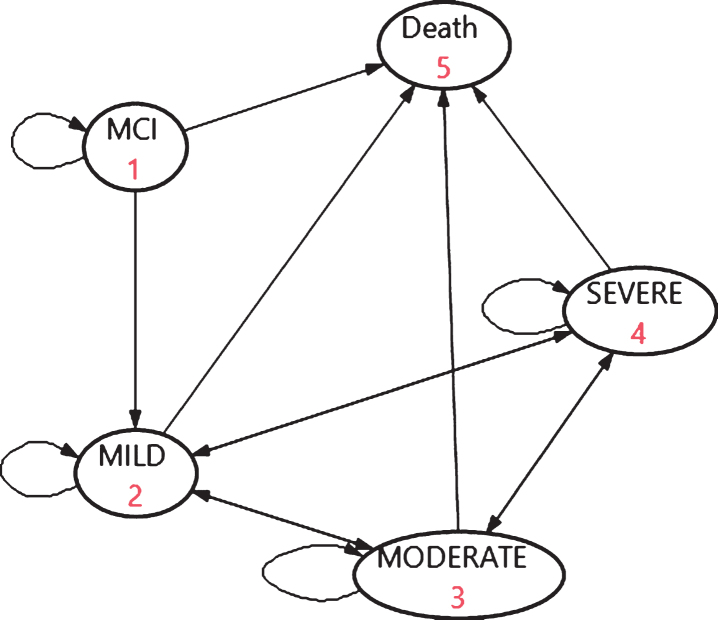
The basic structure of the dementia progression model.

The Markov model with five states (Fig. 1) simulated a virtual cohort of 100,000 people with AD-MCI and the progression within dementia over lifetime. Although 100,000 is hypothetical, it is a rather relevant guess that this figure, based on epidemiology of MCI [19], reflects the magnitude of the numbers of people with AD-MCI in Sweden. In the base case, the start age for the model was 60 with a simulation until age of 100 to reflect the lifetime period, in 40 cycles of 1 year with half cycle correction. Input parameters for disease progression and mortality were derived from SveDem [22]. The assumptions for the base case are presented in Supplementary Table 1. The input estimates represent a Swedish setting, reflecting care in a “Northern Europe welfare state”.

Costs and outcomes (QALYs, see below) represent net present values (NPV) at the cost level of 2016, applying a discount rate of 3% [23]. All costs are expressed as SEK 2016 where 1€= 9.47 SEK and 1 US$ = 8.56 SEK [24]. Effectiveness is expressed in terms of EQ-5D-3L utility-based Quality Adjusted Life Years (QALYs) [25]. The model programming was done in the softwares Treeage® and MS Excel®.

### Risk of progression from AD-MCI to AD-dementia

The uncertainties in AD-MCI progression to AD can be summarized as follows:

#### Uncertainty in the progression risk

Unspecified MCI has been shown in the literature to have a relatively low risk of progression to dementia [18], while AD-MCI has a relatively high risk of progression to AD-dementia [19]. Vos et al. [19] focused on biomarker supported AD diagnoses in MCI in a memory clinic setting. In their review, the 3-year progression to AD dementia varied depending on which diagnostic criteria was used: International working group-1 (IWG-1) [13]: 50%; IWG-2 [14]: 61%; NIA-AA [9]: 5–59%; 59% for the AD high likelihood group. Based on this review, we assume a 3-year progression risk from AD-MCI to AD-dementia of 50% in the base case.

Since our model has 1-year cycles, we assumed a constant progression risk and transformed the 3-year risk to an annual risk using the following formula:

(1) $$p_{1\mathrm{year}}\;=\;1-(1-p_{3\mathrm{year}}{)}^{1/3}$$

where p is the progression to AD dementia risk, which results in an annual risk of 20.6%.

#### Time-dependency in progression rate

For unspecified MCI, the progression to dementia rate decreases over time with a relatively higher rate in the beginning [18], declining to a very small risk after 10 years. For AD-MCI the progression rate is not as clear. If we would have a perfect biomarker supported AD-MCI diagnosis, then the uncertainty around progression to AD-dementia could be reduced significantly. Ad hoc, in the sensitivity analysis of the base case we test the impact of a 10% lower risk for each year in the model, starting from 20.6% as in the base case, to 18.5% in the second year etc.

#### Increased progression risk by age

Since the risk of dementia increases by age, it might be logical to consider age as a risk factor for progression rate. However, this was not the case in the review by Bruscoli [20]. Age might be a risk of having MCI, but not for progression from MCI to dementia, and hence this option is not included in the analyses.

### Transition probabilities

Transition probabilities (TPs) in the model between health states and to death were estimated based on disease progression data contained in the SveDem database [22]. Cognitive impairment severity was classified as Mild (Mini-Mental State Examination (MMSE) 21 to 30), Moderate (MMSE 10 to 20), or Severe (MMSE 0 to 9). Data with possible errors were omitted. These included observations with an interval time smaller than 4 months, individuals without a baseline assessment, observations with duplicate date, individuals with a missing baseline date, individuals with a follow-up before the baseline assessment, individuals with a follow-up date after death, and individuals without any MMSE assessment [26]. Next, we linearly inter- or extrapolated (max. 3 months into the future) MMSE on an annual time grid creating 23,146 annualized transitions between cognitive states (13,394 baseline, 5,477 1-year follow-up, 2,501 2-year follow-up, 1,072 3-year follow-up, 460 4-year; 168 5-year; 61 6-year, and 13 7-year). We took time-dependency into account by using age as a predictor as we believe transition probabilities are dependent on age rather than time since diagnosis because reasons for diagnosis may vary at various stages of disease severity. The analysis, however, indicated that the probability of transition was independent from age, except when we categorized age as higher/lower than 75. An ordered probit regression model was fit to the cognitive states, and the 1-year previous cognitive state was included as covariate (see Supplementary Table 2). We applied inverse probability of censoring weights to adjust for selective drop-out [26].

This model was used to predict the 1-year TPs between the dementia states (Supplementary Table 3).

As seen in Fig. 1, the model assumed transitions from AD-MCI to AD-mild (or remain in AD-MCI or to death), but not to the other dementia states.

### Mortality

Age- and severity-specific transition probabilities to death were based on SveDem and analyzed in two ways. Age specific mortality for people with AD-MCI was assumed to be the same as for the general population and was derived from Statistics Sweden [27]. A Weibull parametric survival regression model was fit to the SveDem data to obtain hazard ratios for Mild, Moderate, and Severe AD-dementia compared to Very mild AD-dementia (MMSE 27 to 30).

Age was used as the time scale on which to model mortality. See Supplementary Table 4 for details. The hazard ratios were applied to the annual age-specific general population mortality rates from the http://www.statistikdatabasen.scb.se (using the formula 1-exp(-age specific annual death probability * HR). Observations were censored 1 year after the last available MMSE status and data from individuals aged younger than 60 were omitted. This resulted in age- and severity-specific TPs to death, used in the base case.

In a sensitivity analysis, a similar approach as in the main analysis was used, except “Very mild” was combined with “Mild dementia” in SveDem in a Weibull parametric survival model. The one-year age- and severity-specific probability of death was predicted using the parametric survival model formula. The two mortality scenarios are shown in Supplementary Figure 1. After about 20 years, about 50% is alive with the base option, while about 2/3 of the simulated cohort is alive in the low mortality option.

### Progression and survival scenarios

Based on the uncertainties, we have modelled three scenarios: 1) Non-time dependent progression (base case); 2) Time-dependent progression (sensitivity analysis); and 3) Non-time dependent progression (as option 1), but lower mortality scenario (sensitivity analysis).

### Resources and costs inputs

Societal costs were derived from a population based Swedish costing database that has been used in several studies [28–30]. Costs are expressed in terms of cognitive status (normal, MCI, and Mild, Moderate, and Severe dementia), type of dementia, and age. The most important cost drivers in dementia care are included: living situation (such as at home or institutional care), social services at home, hospital care, drug costs, and informal care. Unit costs are shown in Supplementary Table 5. These cost items constitute about 94% of the societal costs [31]. A log link Generalized Linear Model (GLM) with a gamma distribution was used to describe costs in relation to age and level of cognitive impairment (Supplementary Figure 2).

### Outcomes: Quality adjusted life years (QALYs)

Health-related quality of life utility estimates were obtained from EQ-5D-3L [32]. For the dementia states (Mild-Moderate-Severe), a Swedish study on dementia was used [33]. To get utilities for AD-MCI, two sources were used [25, 34]. Based on the linear relation between age and the EQ-5D-3L utilities in the Burström paper with an age span 20–88 years [25] and their further regression analyses [35, 36], we estimated a similar a linear regression (QALY = –0.002464 * age + 0.962679 (a start age of 25), where it was also assumed that the same change in QALY per 5 years age classes was relevant also for AD-dementia (Supplementary Table 6). For correlation between the EQ5D-3L values in the core paper and predicted values, see Supplementary Table 7.

### The hypothetical illustrative intervention

So far, no DMT has shown significant efficacy in a trial. Thus, any model must be based on hypothesized speculative intervention effects. The term “disease modifying” likely does not imply total cure or complete stop of disease progression in AD. Also, we do not know when to start treatment and when to stop treatment as its effectiveness and possible side effects might differ across states. We therefore test different hypothetical intervention effects, different treatment duration, and different treatment start to illustrate various potential future scenarios.

In the base case intervention (BI), we assume the hypothetical DMT starts in AD-MCI, reduces the risk for progression to AD-dementia by 25%, and reduces the progression rate within Mild AD-dementia by 25%. The treatment with a DMT is assumed to take place during the MCI-AD and mild AD-dementia states. The start age for DMT is 60 and the duration is 40 years.

Since we have no DMT on the market, there is no price. Therefore, we used a hypothetical “anchor price” of 50,000 SEK per year for a DMT. However, this is not a price recommendation; it is used just for illustrative purposes in the model. The potential incremental cost effectiveness ratio (ICER), net monetary benefits (NMB), and costs per person year (PY) without dementia were used as cost effectiveness measures. Further, the potential differences in cumulative cases with dementia and deaths as well as differences in PYs in states and numbers needed to treat (NNT) to avoid one case of dementia were also presented.

### Sensitivity analysis of intervention

The following scenarios were tested in the one-way sensitivity analyses (for details, see Supplementary Tables 8 and 9):

1) Different intervention effects: The rationale for these sensitivities is to test different magnitudes of intervention effects, but also different starting points and treatment durations (Sensitivity 1–7 of base intervention).
2) Different inputs: The rationale for these sensitivities is to test how various uncertainties in inputs influence the robustness of the results (Sensitivity 8–11 of base intervention).
3) Different discount rates (base case 3%): 1% and 5% (Sensitivity 12-13 of base intervention).

In a probabilistic sensitivity analysis (PSA) of base intervention, the most relevant factors for uncertainty were included: the cost of the intervention, the risk of conversion to dementia, progression rate, state related QALYs, costs, and mortality. Furthermore, we assumed 25% of the mean as the standard error around the mean. For the costs, gamma distributions were applied. The parameters in the PSA were not age-dependent and were derived from the age of 71, where the ICER was > 99% in line with the base case. The PSA was run with 1,000 iterations.

## RESULTS

### Base case: Survival and disease progression

**Fig. 2:**
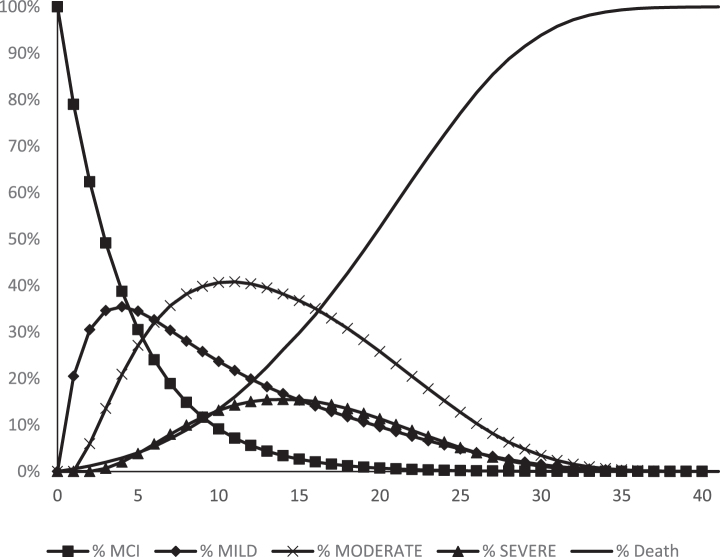
The course of the simulated cohort during the simulated period of 40 years. Year 0 = start age 60.

After 20 years, about half of the simulated cohort have reached the state “Death” (Fig. 2) and at the end of the modelling period, almost everyone is dead. Furthermore, the cumulative risk to develop AD-dementia with the fixed annual risk of ∼20% is 96%, which means almost no survived persons in the simulated cohort terminates the model in the state of AD-MCI. The greatest area under the curve (AUC) is in the Moderate dementia state, which is where most of the cohort’s PYs were concentrated. About 78% of the cumulative PYs were spent in an AD-dementia state (Table 1).

**Table 1:** Main outcomes in the simulated cohort of 100,000 MCI-AD persons after 40 years

	10 years	20 years	30 years	40 years
Cumulative cases of dementia	89,607	95,573	95,957	95,969
Cumulative deaths	13,391	52,501	93,908	99,987
Cumulative deaths with dementia	10,275	48,647	89,892	95,957
Cumulative deaths with AD-MCI	3,116	3,854	4,016	4,030
Mean PYs/person	10.36	16.86	18.88	18.98
Mean PYs/person with dementia	6.44	12.68	14.68	14.78
Mean PYs/person AD-MCI (without dementia)	3.92	4.18	4.20	4.20
Mean PYs/person in Mild AD	3.07	4.51	4.94	4.97
Mean PYs/person in Moderate AD	2.74	6.14	7.26	7.31
Mean PYs/person in Severe AD	0.62	2.03	2.48	2.50

PYs, person years.

The cumulative total healthcare resource cost was 450 billion SEK (53 billion US$), with the highest cost, about 189 billion SEK (22 billion US$) in Moderate AD (Table 2). By year 5, AD-MCI and AD-dementia costs are about the same. Between years 10 and 20, the cumulative costs increased considerably, and then survival effects (deaths increase) made cumulative costs increase slower. Between years 30 and 40, very little happens in the model since most people already have died. The average cost for a PY with dementia was 252,843 SEK (29,538 US$).

**Table 2:** Cumulative cohort cost distribution (state related cumulative costs, SEK) and QALY distribution (state related cumulative) (both NPVs) during the simulated period (1 US$ = 8.56 SEK)

Severity state	5 years	10 years	20 years	30 years	40 years
Costs (million SEK)
AD-MCI	58,050	71,688	76,474	76,762	76,771
Mild	35,482	65,823	95,854	104,153	104,650
Moderate	19,624	71,216	160,064	188,121	189,440
Severe	2,373	19,222	64,610	79,051	79,514
Total costs	115,528	227,949	397,002	448,087	450,375
Cost/PY AD-dementia (SEK)	216,937	242,747	252,740	252,864	252,843
QALYs
AD-MCI	235,736	285,845	301,365	302,127	302,146
Mild	95,364	169,241	232,408	246,675	247,384
Moderate	25,665	87,034	176,846	199,734	200,606
Severe	1,569	11,598	34,065	39,678	39,819
Total QALYs	358,334	553,718	744,683	788,214	789,956

The largest proportion of the cumulative cohort QALYs occur in the AD-MCI state, but due to survival and progression effects, the cumulated QALYs increase much more in the other states (Table 2). See NPVs of state specific costs and QALYs in Supplementary Tables 10 and 11.

### Sensitivity analysis of the base case

The greatest differences occurred in the number of dementia cases and PYs with dementia between the fixed and risk reduction scenarios (Table 3). With the risk reduction option, the time (PYs) in AD-MCI was about 2.7 years longer. The total costs at the end of the simulation did not differ so much, but the distribution of costs differed with higher costs in AD-MCI and lower costs in dementia states in the risk reduction scenario.

**Table 3:** Summary of the sensitivity analysis of the base case in relation to severity states when appropriate (1 US$ = 8.56 SEK). Time horizon 40 years. Costs and QALYs as NPVs

	AD-MCI	Mild	Moderate	Severe	All
**Cumulative deaths: Base case**					**99,987**
Cumulative deaths: High risk, risk reduction					99,549
Cumulative deaths: Lower mortality					97,078
**Cumulative cases of dementia: Base case**					**95,969**
Cumulative cases of dementia: High risk, risk reduction					83,661
Cumulative cases of dementia: Lower mortality					95,969
**Cohort results (mSEK)**
**Cohort costs: Base case**	**76,771**	**104,650**	**189,440**	**79,514**	**450,375**
Cohort costs: High risk, risk reduction	118,883	90,962	164,723	69,108	443,676
Cohort costs: Lower mortality	76,771	119,196	246,956	115,951	558,874
Cohort costs: Discount rate 1%	83,012	127,525	248,538	107,598	566,673
Cohort costs: Discount rate 5%	71,498	87,675	147,456	59,863	366,492
**Cohort QALYs: Base case**	**302,146**	**247,384**	**200,606**	**39,819**	**789,956**
Cohort QALYs: High risk, risk reduction	436,308	215,179	174,779	34,711	860,977
Cohort QALYs: Lower mortality	302,146	272,889	248,402	54,375	877,812
Cohort QALYs: Discount rate 1%	324,990	295,802	258,086	52,897	931,775
Cohort QALYs: Discount rate 5%	282,652	210,677	158,976	30,500	682,805
**Person years (PYs) results**
**PYs: Base case**	**4.20**	**4.97**	**7.31**	**2.50**	**18.98**
PYs: High risk, risk reduction	6.93	4.32	6.35	2.17	19.77
PYs: Lower mortality	4.20	5.78	9.83	3.76	23.57

### Potential results of the hypothetical base intervention

The hypothetical intervention resulted in potentially 2,447 fewer cases with dementia over 40 years (Table 4). The greatest effect on NNTs occurred in the first decade. In terms of PYs, people live 1.44 PYs less with dementia (they stay longer in AD-MCI). The hypothetical intervention also resulted in potentially increased costs but also potentially more gained QALYs, resulting in an ICER of 532,519 SEK (62,210 US$) per gained QALY (Table 5), which is lower than the assumed willingness to pay (WTP) level of 600,000 SEK (70,000 US$), supporting cost effectiveness. The cut off treatment price for absolute cost savings (i.e., budget neutral situation) was 7,155 SEK/year (836 US$/year) and for a WTP of 600,000 SEK, the threshold price is 55,429 SEK/year (6,475 US$/year). The relations between different WTP levels and threshold prices of the hypothetical DMT are seen in Supplementary Figure 3. The potential cost per avoided PY with dementia was 271,054 SEK (31,655 US$).

**Table 4:** Epidemiological outcomes of the base intervention of a simulated cohort of 100,000 persons with AD-MCI

	No intervention	Intervention	Difference
Cumulative cases with dementia	95,969	93,523	–2,447
Cumulative deaths	99,987	99,969	–19
Cumulative deaths with dementia	95,957	93,495	–2,462
Cumulative deaths with AD-MCI	4,030	6,473	2,443
PYs/person (survival years)	18.98	19.63	0.65
PYs/person with dementia	14.78	13.99	–0.79
PYs/person AD-MCI (without dementia)	4.20	5.64	1.44
PYs/person in Mild AD	4.97	5.83	0.86
PYs/person in Moderate AD	7.31	6.14	–1.18
PYs/person in Severe AD	2.50	2.02	–0.47
NNT to avoid one case of dementia in 10 years			12
NNT in 20 years			29
NNT in 30 years			40
NNT in 40 years			41
NNT to avoid one case of death			5,388

PYs, person years; NNT, numbers needed to treat.

**Table 5:** Cost effectiveness of the base intervention (BI) (1 US$ = 8.56 SEK). Costs and QALYs as NPVs

	No intervention	Intervention	Incrementals
Cost of intervention: 50,000 SEK
Costs	4,503,751	4,893,703	389,952
QALYs	7.90	8.63	0.73
ICER			532,519
NMB at WTP (600,000 SEK)			48,048
Cost/PY without dementia			271,054
Cost per prolonged PY			596,994

QALYs, Quality Adjusted Life Years; ICER, incremental cost effectiveness ratio; NMB, net monetary benefits; WTP, willingness to pay; PY, person year.

### Sensitivity analysis of the base intervention

Supplementary Tables 8 and 9 summarizes the sensitivity analyses of the intervention. In general, all options show a potentially reduced number of people with dementia by the hypothetical treatment. No absolute cost savings occur. In the PSA, the probability of cost effectiveness was 61.3% (acceptability curve for different levels of WTP, see Supplementary Figure 4).

## DISCUSSION

### The results of the base option

The lifetime risk of developing AD-dementia from AD-MCI is very high. 96% developed AD-dementia during the period. This is in line with the outcome with a biomarker supported AD diagnosis. In the 20 years scenario, 95% had already developed AD-dementia even though about 50% still were alive. The death rates are high as expected. The costs were high for mild cases, which reflects that the cost of care for the elderly are relatively high in Sweden as compared to many other countries [31]. Nevertheless, there are different costs across different levels of cognitive capacity (the states), making the analysis of hypothetical interventions interesting.

### The hypothetical intervention

It is unrealistic to assume a hypothetical future DMT for AD would result in absolute cost savings because of the cost associated with the treatment and prolonged survival.

If the cost of a hypothetical DMT was set to 0, there would be absolute cost savings, about 65,000 SEK/person (7,600 US$/person). This figure is perhaps smaller than expected, but since the hypothetically treated persons are expected to live longer, this potential effect results in higher care costs in the treated group. The appropriate approach should be lifetime cost effectiveness in terms of societal WTP, which for pharmaceutical products are heavily linked to reimbursement issues.

An early treatment start (in AD-MCI) is potentially better than a late start (in Mild AD dementia). In terms of PYs in different states, the period in early states (AD-MCI and Mild AD) is longer and the period in late states (Moderate and Severe) is shorter in base intervention option than it is in sensitivity 1 (start in Mild AD-dementia). Starting the hypothetical DMT later resulted in higher incremental costs and less benefits in terms of QALYs, and thus giving a higher ICER compared to early treatment start.

The efficacy of the hypothetical treatment directly affected its ability to demonstrate potential cost-effectiveness. With the 25% reduction in progression in AD-MCI and Mild AD dementia, the ICER was rather close to the assumed WTP, but with 50% effect, results were more convincing, although not cost saving. The case with a hypothetical total stop of progression to AD-dementia seems hardly realistic, but illustrates the potential of a “wonder drug” for AD. To start at age 70 (with a time horizon of 30 years) results in potentially more avoided cases of dementia and a lower NNT than to start at age 60, given the same size of the starting population. The ICER is somewhat higher for a later start, but these differences seem less significant as compared to the better clinical outcomes.

The potential NNT (and its associated cost per PY) to avoid one case of dementia can be discussed vs the efficacy levels. Here, it appears that a 50% risk reduction is a wishful goal.

### Methodological issues

#### Progression to dementia risk

Crucial for any economic simulation in AD is the risk of progression to AD-dementia from predementia states. The use of different biomarkers has improved these risk estimates. Unspecific MCI is a very heterogeneous phenomenon, which is of interest in public health prevention discussions, but for AD specifically the narrower concept AD-MCI is more appropriate. We also used a fixed progression to dementia figure and not an age-related one. Age is of course a risk factor for having MCI, but not necessarily for progression to AD-dementia for AD-MCI [20].

How the progression risk from AD-MCI to AD-dementia changes by time, given the same start age is another issue for discussion. However, as shown in the sensitivity analysis, the difference between a fixed risk and a decreasing risk (sensitivity 8), was small in the cost effectiveness analysis.

#### Disease progression

The TPs used in this model are based on the SveDem database. The TP option “unchanged” was rather high in all states. This is the case in most datasets used for simulations, but somewhat higher here, making the magnitude for intervention effects smaller. However, SveDem is a very large database and probably is well representative for the “real world”.

The simulations indicate that continuing the hypothetical treatment beyond Mild AD may potentially be less cost effective, even if the treatment is effective. The highest cohort cost also occurs in Moderate AD. Whether treatment should stop due to low cost effectiveness at any disease stage cannot be answered in a model like this with fixed states. Individual microsimulation can perhaps address this issue better.

In the base intervention, backward transitions occurred. Although this should not be the case in theory, it often is observed in “real world”, such as in SveDem. In the sensitivity No 10 we tested an option without backward transitions. It resulted in somewhat better cost-effectiveness, but the difference versus the base intervention was small.

#### Mortality

In this paper, we used two ways of estimating mortality. In the base option, mortality was calculated by multiplying the relative risk of Mild, Moderate, and Severe dementia to the estimates of annual age-specific probability of death provided by Statistics Sweden. This, however, likely overestimates death in higher ages as the prevalence of dementia increases with age and is represented increasingly with age.

For these reasons, alternatively mortality estimates were based only on the observed survival from the SveDem data. The SveDem data is linked to a register of complete death dates [37]. However, part of the time between the last observation of cognition and death was missing. This omission was likely conditional on a worse cognitive status and likely underestimates mortality in severe stages. Furthermore, it is likely that those with more comorbidities are more likely to drop-out, resulting in an underestimation of mortality at higher ages. It is difficult to estimate the magnitude of the bias and therefore the two options likely represent the uncertainty interval of the effect of dementia on death. The different mortality scenarios (base intervention and sensitivity 9) had an effect on the magnitudes of the results, but not so much on the increments.

#### AD diagnoses

It has been suggested in several consensus documents that a diagnosis of AD needs to be confirmed by imaging and biomarkers [8, 9, 38, 39]. In SveDem, about 92% at memory clinics and 84% in primary care undergo a CT scan, 19% and 3% undergo a MR investigation. At memory clinics 45% also go through a CSF investigation [40]. However, it is not required that an AD diagnosis in SveDem should be based on imaging or biomarkers. The consensus documents also focus on research criteria, and it is not clear how to use them in clinical practice [41].

SveDem is by definition a registry for people with a diagnosed dementia disorder in clinical practice. There are people in the registry in the MMSE interval 27–30 with a diagnosis of dementia. Although such people often are labelled as MCI, there are people with dementia that may present MMSE between 27 and 30, and these people are here labelled as “Very mild dementia”, although it may be difficult to surely say whether they have dementia or MCI.

#### Outcomes

Parallel to the traditional health economic outcomes (QALYs, ICER, NMB), we also present a set of clinical and epidemiological outcomes, such as survival patterns, distributions in different states during the simulated periods, and NNTs, that can be valued versus costs. Particularly for long lasting chronic disorders, where a slowing down in deterioration may be regarded as a positive effect, we regard the clinical and epidemiological outcomes as a valuable complement in health economic analyses.

### Limitations

Besides the methodological challenges that we have discussed above, the greatest limitation is the intervention’s hypothetical shape by applying an assumed speculative treatment effect on costs and outcomes. The value of this illustration is in adding to the methodological development and discussing the potential impact to support considerations related to target population, expected extrapolated long-term effects, and budget impact. This model is limited in the translation of trial outcomes (typically memory scales after 12–24 months) to a relative risk of progression to AD-dementia, which is challenged by assumptions on causality and long-term duration or extrapolation of treatment effect.

Regarding the QALYs, we assume that the age dependent relationships for a normal aging population as presented by Burström et al. [25], also have the same slope in different severity states of AD. This assumption needs to be discussed and analyzed in specific studies on AD.

The generalizability of the SveDem registry is limited due to missing data [26] and being specific to Sweden, but strengthened by its clinical setting.

The potential impact of an intervention is limited to the subpopulation of people with dementia, who are identified by the care system and labelled as AD-MCI. People with other-type MCI might progress to AD-dementia, but are not reflected in this analysis.

Future studies could estimate the value of perfect information, for example on the effect of age and time since diagnosis of AD-MCI on progression to AD-dementia, reduced missing data in the SveDem registry, and estimates of mortality.

Most economic simulations in AD have an empirical core of an existing intervention effect (drug, psychosocial interventions, prevention, etc.). This is not the case with our model. This situation is a consequence of the fact that there is so far no DMT available with a set price and a shown effect on disease progression. Economic simulations per se are based on a set of assumptions (disease progression, survival, inputs for costs and outcome, etc.) which may be questionable and the lack of empirical data on interventions make our model even more problematic. However, emergence of a DMT is still possible (which probably will be much more expensive than current drugs), and hence it is important to understand the potential impact of such a treatment on scarce societal resources. Further research in basic AD research, drug development as well as generation of new data on disease progression and modelling techniques should be given priorities.

### Conclusions

SveDem is a powerful tool for health economic analyses. Lifetimes societal costs of AD are substantial. Based on this model, absolute cost savings are not likely from a hypothetical DMT, but it could be a potentially cost effective option to prevent AD-dementia. At least 25-50% slowing of disease progression would results in favorable epidemiological and health-economic outcomes. Starting treatment in earlier states (AD-MCI) is potentially favorable compared to a later state (Mild AD-dementia).

## Supplementary Material

Supplementary Material
