# Supplementary material for: Quantifying and Describing the Natural History and Costs of Alzheimer’s Disease and Effects of Hypothetical Interventions
Source: J Alzheimers Dis. 2020 Jun 2;75(3):891–902. doi: 10.3233/JAD-191055 (PMC7369101; doi:10.3233/JAD-191055)
Supplement: Supplementary Material [file jad-75-jad191055-s001.pdf]

Supplementary Material

Quantifying and Describing the Natural History and Costs of Alzheimer's Disease and Effects of Hypothetical Interventions

Supplementary Table 1. Assumptions for the base case (1 US$ = 8.56 SEK).

Assumption	Value	Source
Year of cost	2016	-
Cycles	40	-
Cycle length	1 year	-
Start age	60	-
Discount rate costs and QALYs	3%	-
Cohort size	100,000	-
AD-MCI progression risk to AD-dementia	Not age dependent	[1, 2], SVEDEM
Transition probabilities	Not age dependent	SVEDEM
AD survival	Age dependent	SVEDEM
Costs	Age dependent	[3-5]
QALYs	Age dependent	[6, 7]
Diagnostic cost in primary care	7,000 SEK	[8]
Diagnostic cost in primary care	12,000 SEK	[8]

Supplementary Table 2. Results of the ordered probit analysis in SveDem (dependent: categorized MMSE mild, moderate, severe; based on 23,146 observations from 13,445 individuals).

Parameter	Coefficient	95% confidence interval	p
MMSE categorized			
Mild (MMSE 21-30)	reference		
Moderate (MMSE 10-20)	1.8984	1.8479 to 1.949	<0.001
Severe (MMSE 0-9)	3.9837	3.7343 to 4.233	<0.001
Diagnosis			
Alzheimer's	reference		
Other dementia	-0.116	-0.179 to -0.054	<0.001
Unspecified	-0.025	-0.086 to 0.036	0.418
Cut 1	0.542	0.510 to 0.574	
Cut 2	3.129	3.052 to 3.206	

A selection was made on available transitions (i.e., requiring 2 inter- or extrapolated observations (extrapolation maximum of 3 months into the future). The SveDem obtained 53,880 baseline assessments and 37,491 follow-up assessments of which 25,436 had an MMSE available (68%) (see Handels et al. [9]).

Supplementary Table 3. Predicted transition probabilities for AD states based on the ordered probit model coefficients provided in Supplementary Table 2.

		to:		
	States	Mild	Moderate	Severe
from:	Mild	0.706	0.293	0.001
	Moderate	0.087	0.803	0.109
	Severe	0.000	0.196	0.804

Supplementary Table 4. Results of the survival analysis in SveDem registry (52,969 subjects*; 4,674 deaths; Weibull distribution).

**Parameter**	**Coefficient**	**95% confidence interval**	**p**
MMSE categorized			
Very mild (MMSE 27-30)	reference		
Mild (MMSE 21-26)	1.318	1.153 to 1.507	<0.001
Moderate (MMSE 10-20)	2.419	2.122 to 2.757	<0.001
Severe (MMSE 0-9)	4.267	3.610 to 5.043	<0.001
Diagnosis			
Alzheimer's	reference		
Other dementia	1.775	1.657 to 1.902	<0.001
Unspecified	1.200	1.119 to 1.286	<0.001
Constant	0.000	0.000 to 0.000	<0.001
Weibull shape parameter p	7.546	7.166 to 7.946	

*This analysis was based on the subsample of persons 60 years or older (n=53,018). A small proportion of this sub-population (<0.1%) had missing MMSE and could not be used for the analysis. Note the difference from the sample used for the ordered probit, which required at least 2 inter- or extrapolated MMSE observations.

Supplementary Table 5. Unit costs (SEK, 2016 price level) (1 US$ = 8.56 SEK).

	Unit cost	Source
Long term institutional care (per day)*	1,880	https://skl.se/ekonomijuridikstatistik/statistik/kostnadperbrukarekpb/aretsjamforelseavkpbnyckeltal.803.html
Hospital care (per day)	6,572	https://statva.skl.se/SASVisualAnalyticsViewer/guest.jsp?reportPath=/Web/Prod/Rapporter/Publik/KPP/&reportName=KPP%20SV%20Publik%20DRG%20alder%20kon&commentsEnabled=false&appSwitcherDisabled=true&propertiesEnabled=false&reportViewOnly=true
Home services (per hour)	472	https://skl.se/ekonomijuridikstatistik/statistik/kostnadperbrukarekpb/aretsjamforelseavkpbnyckeltal.803.html
Informal care (per hour)	169	http://www.statistikdatabasen.scb.se/pxweb/sv/ssd/START__AM__AM0110__AM0110A/LonYrkeRegion4A/table/tableViewLayout1/?rxid=d14d23a1-c6f6-46b1-96e1-2e38882d78b2
Drugs (per person and year)	Community: 5,701<br>Institution: 7,816	[10]

*Costs are adjusted for so called “hotel costs” (costs for housing and food).

Supplementary Table 6. Age specific EQ-5D-3L utilities.

Age	AD-MCI	Mild	Moderate	Severe
50	0.839	0.706	0.462	0.309
55	0.827	0.694	0.450	0.296
60	0.815	0.681	0.438	0.284
65	0.803	0.669	0.425	0.272
70	0.790	0.657	0.413	0.259
75	0.778	0.644	0.401	0.247
80	0.766	0.632	0.389	0.235
85	0.753	0.620	0.376	0.223
90	0.741	0.607	0.364	0.210
95	0.729	0.595	0.352	0.198
100	0.716	0.583	0.339	0.186

Supplementary Table 7. Correlation between EQ-5D values from source paper and predicted values based on regression formula.

Age	EQ-5D	Predicted (age class mitt)
50-59	0.83	0.83
60-69	0.80	0.80
70-79	0.79	0.78
80-88	0.74	0.75

Supplementary Table 8. Sensitivity analyses: The differences between intervention and no intervention for the epidemiological outcomes.

Epidemiological outcomes	BI	Sens 1	Sens 2	Sens 3	Sens 4	Sens 5	Sens 6	Sens 7	Sens 8	Sens 9	Sens 10	Sens 11
Cumulative cases with dementia	-2,447	NA	-2,447	-2,447	-4,890	-3,394	-8,519	-95,969	-9,346	-2,447	-2,447	-1,949
Cumulative deaths	-19	-11	-8	-23	-60	-3,673	-115	-3,639	-330	-615	-9	-3
Cumulative deaths with dementia	-2,462	NA	-2,451	-2,465	-4,930	-5,561	-8,592	-95,597	-9,356	-3,058	-2,452	-425
Cumulative deaths with AD-MCI	2,443	NA	2,443	2,443	4,871	1,888	8,477	92,317	9,026	2,443	2,443	422
PYs/person (survival years)	0.65	0.57	0.36	0.82	0.59	0.36	1.71	5.99	0.90	0.48	0.74	0.28
PYs/person with dementia	-0.79	-0.57	-1.08	-0.62	-0.62	-1.02	-2.21	-14.78	-1.51	-0.96	-0.70	-0.28
PYs/person AD-MCI (without dementia)	1.44	NA	1.44	1.44	1.21	1.38	3.92	20.76	2.41	1.44	1.44	0.56
PYs/person in Mild AD	0.86	1.62	-0.26	0.99	0.44	0.64	1.80	-4.97	0.37	1.09	0.80	0.75
PYs/person in Moderate AD	-1.18	-0.11	-0.58	-0.73	-0.81	-1.19	-2.89	-7.31	-1.36	-1.41	-0.64	-0.86
PYs/person in Severe AD	-0.47	-0.94	-0.24	-0.88	-0.24	-0.46	-1.11	-2.50	-0.51	-0.64	-0.86	-0.17
NNT to avoid one case of dementia	41	NA	41	41	20	29	12	1	11	41	41	51

BI. Treatment in AD-MCI and Mild AD, intervention 25% risk reduction of converting to AD dementia from AD-MCI, fixed rate of conversion, start age 60, time horizon 40 years, high mortality, discount rate 3.5%.
Sensitivity 1. Treatment during two states as above but with late start: Mild and Moderate AD (start in Mild AD; no AD-MCI results).
Sensitivity 2. Intervention with 25% risk reduction of converting to AD dementia from AD-MCI (no treatment in Mild AD).
Sensitivity 3. Intervention with 25% risk reduction of converting to AD dementia from AD-MCI and 25% slower progression in Mild and Moderate AD.
Sensitivity 4. Start age 70 and simulation period 30 years.
Sensitivity 5. Time horizon 20 years from start age 60.
Sensitivity 6. Intervention with 50% risk reduction of converting to AD dementia and 50% slower progression in Mild AD, otherwise as BI.
Sensitivity 7. 100% stop of progression to dementia, treatment during AD-MCI.
Sensitivity 8. Decreasing progression rate to dementia, instead of fixed rate.
Sensitivity 9. Lower mortality (in AD-dementia, not in AD-MCI).
Sensitivity 10. No backward transitions in dementia.
Sensitivity 11. SveDem AD-MCI progression rate inputs. The Svedem registry has a subgroup of AD persons with MMSE 27-30, which we labelled as "AD-MCI.

Supplementary Table 9. Sensitivity analyses: summary of the differences between intervention and no intervention for the cost effectiveness. Analyses (intervention cost 50,000 SEK) (1 US$ = 8.56 SEK). Costs and QALYs as NPVs.

	BI	Sens 1	Sens 2	Sens 3	Sens 4	Sens 5	Sens 6	Sens 7	Sens 8	Sens 9	Sens 10	Sens 11	Sens 12	Sens 13
Costs (incrementals)	389,952	584,317	361,605	614,867	371,941	321,930	412,125	382,023	455,630	347,113	331,507	242,747	407,805	384,214
QALYs (incrementals)	0.73	0.52	0.46	0.82	0.60	0.64	1.79	5.44	0.89	0.72	0.80	0.41	0.94	0.58
ICER	532,519	1,121,300	786,676	747,404	624,668	505,744	229,891	70,265	511,300	485,327	416,540	599,695	432,810	659,672
NMB with WTP = 600, 000 SEK	48,048	-271,680	-85,808	-121,265	-14,688	59,998	663,495	288,022	79,042	81,887	146,093	124	157,395	-35,014
Cost/PY without dementia	271,054	NA	251,350	427,391	308,306	233,260	105,126	18,398	188,926	241,273	230,429	436,388	283,463	267,065

BI. Treatment in AD-MCI and Mild AD, intervention 25% risk reduction of converting to AD dementia from AD-MCI, fixed rate of conversion, start age 60, time horizon 40 years, high mortality, discount rate 3.5%.
Sensitivity 1. Treatment during two states as above but with late start: Mild and Moderate AD (start in Mild AD; no AD-MCI results).
Sensitivity 2. Intervention with 25% risk reduction of converting to AD dementia from AD-MCI (no treatment in Mild AD).
Sensitivity 3. Intervention with 25% risk reduction of converting to AD dementia from AD-MCI and 25% slower progression in Mild and Moderate AD.
Sensitivity 4. Start age 70 and simulation period 30 years.
Sensitivity 5. Time horizon 20 years from start age 60.
Sensitivity 6. Intervention with 50% risk reduction of converting to AD dementia and 50% slower progression in Mild AD, otherwise as BI.
Sensitivity 7. 100% stop of progression to dementia, treatment during AD-MCI.
Sensitivity 8. Decreasing progression rate to dementia, instead of fixed rate.
Sensitivity 9. Lower mortality (in AD-dementia, not in AD-MCI).
Sensitivity 10. No backward transitions in dementia.
Sensitivity 11. SveDem AD-MCI progression rate inputs. The Svedem registry has a subgroup of AD persons with MMSE 27-30, which we labelled as "AD-MCI.
Sensitivity 12 Discount rate 1%.
Sensitivity 13. Discount rate 5%.

Supplementary Table 10. Mean cost (SEK) per person and PY per severity state (1 US$ = 8.56 SEK). Costs as NPVs, start age 60.

Severity state	Cost per person and PY(SEK)
AD-MCI	182,831
Mild AD	210,711
Moderate AD	259,043
Severe AD	318,495
All	237,350
AD-dementia	252,843

Supplementary Table 11. QALYs per person and PY (NPVs).

Severity state	QALY
QALYs AD-MCI	0.720
QALYs Mild	0.498
QALYs Moderate	0.274
QALYs Severe	0.159
All	0.416

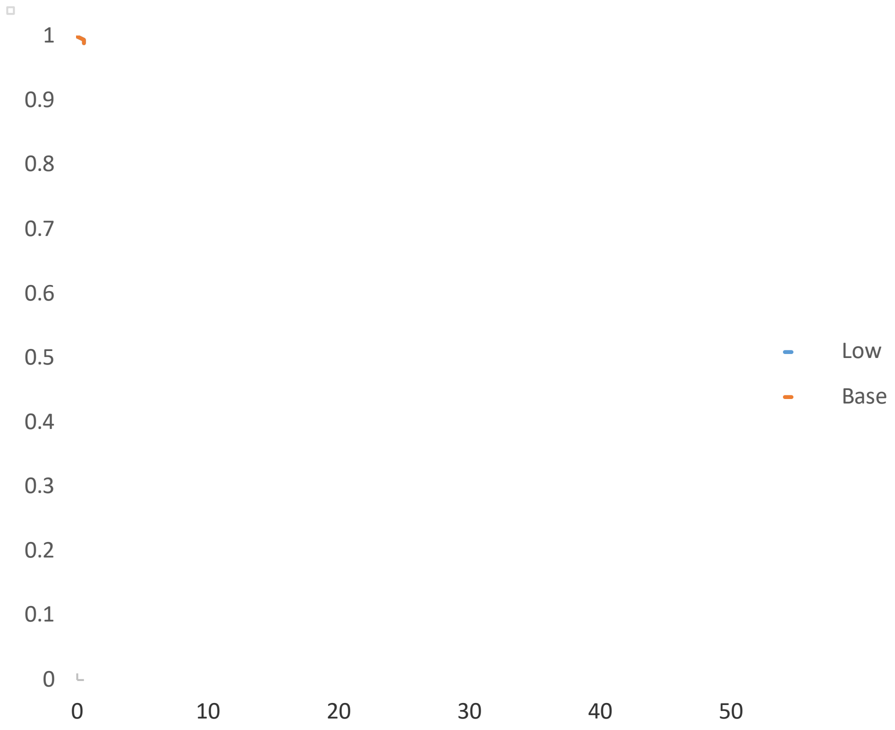

Supplementary Figure 1. Survival curves with the base case and a lower mortality. Year 0 = start age 60.

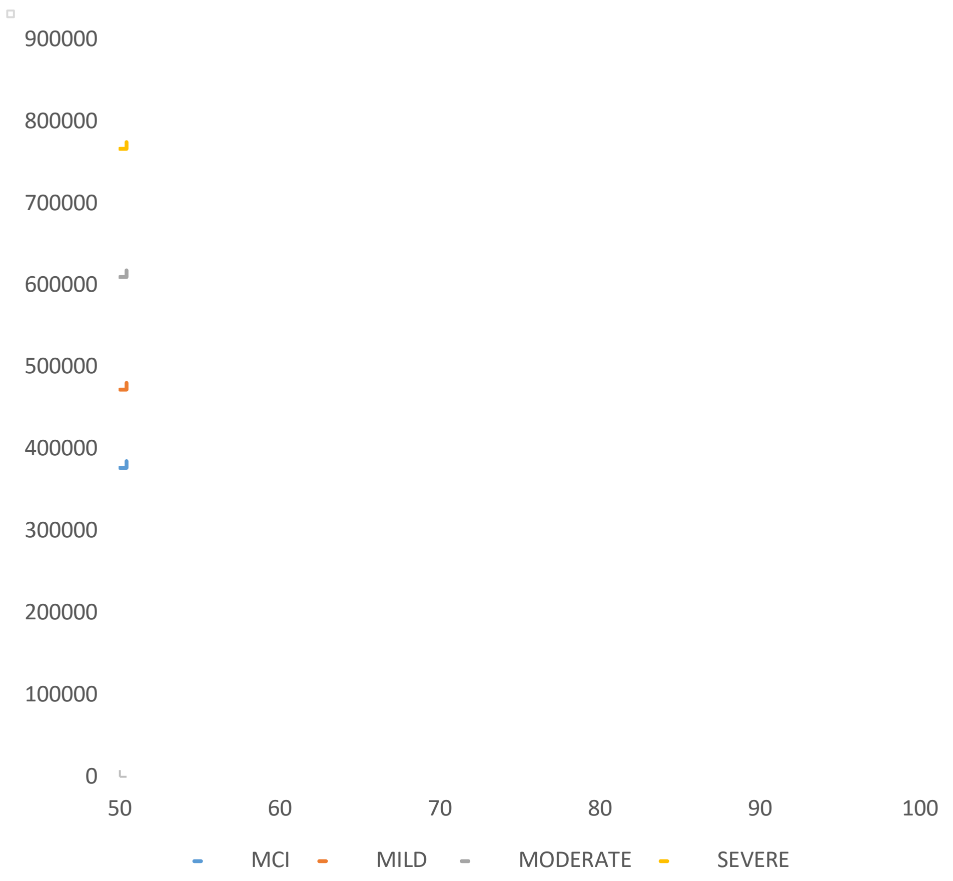

Supplementary Figure 2. GLM of annual societal costs (SEK) (including costs also for comorbidities) of people with different states of cognitive impairment due to AD in relation to age.

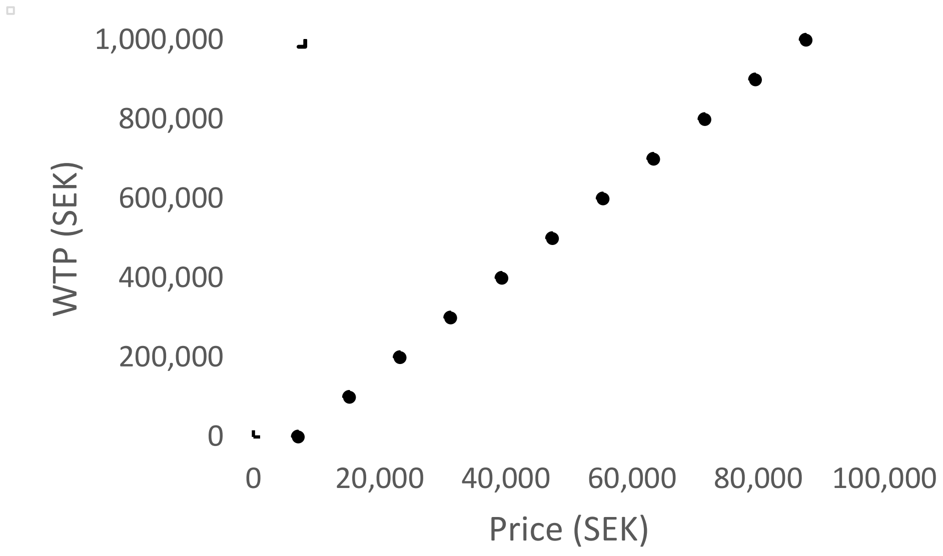

Supplementary Figure 3. Relationship between different WTP levels and price for the DMT for cost-effectiveness (1 US$ = 8.56 SEK).

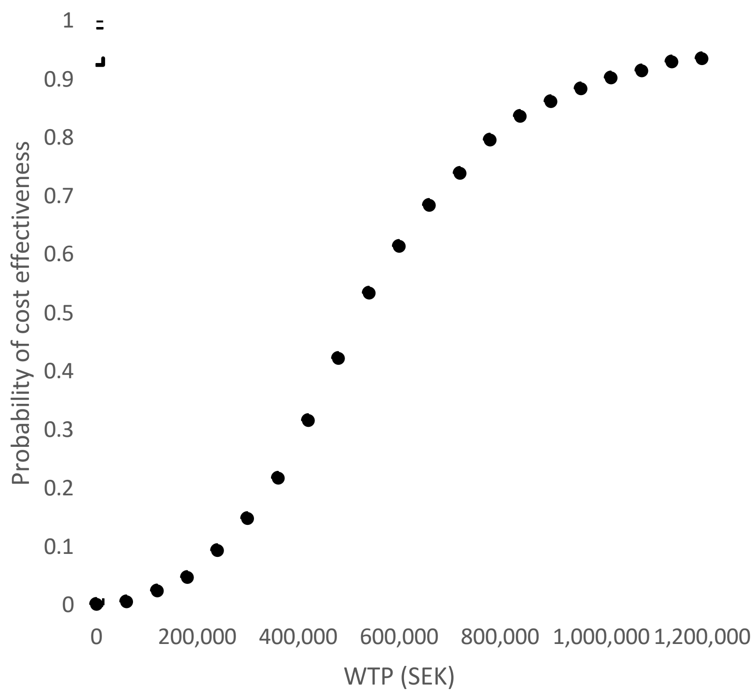

Supplementary Figure 4. Acceptability curve at different levels of WTP (1 US$ = 8.56 SEK).

REFERENCES

[1] Mitchell AJ, Shiri-Feshki M (2009) Rate of progression of mild cognitive impairment to dementia--meta-analysis of 41 robust inception cohort studies. *Acta Psychiatr Scand* **119**, 252-265.

[2] Vos SJ, Verhey F, Frolich L, Kornhuber J, Wiltfang J, Maier W, Peters O, Ruther E, Nobili F, Morbelli S, Frisoni GB, Drzezga A, Didic M, van Berckel BN, Simmons A, Soininen H, Kloszewska I, Mecocci P, Tsolaki M, Vellas B, Lovestone S, Muscio C, Herukka SK, Salmon E, Bastin C, Wallin A, Nordlund A, de Mendonca A, Silva D, Santana I, Lemos R, Engelborghs S, Van der Mussele S, Alzheimer's Disease Neuroimaging Initiative, Freund-Levi Y, Wallin AK, Hampel H, van der Flier W, Scheltens P, Visser PJ (2015) Prevalence and prognosis of Alzheimer's disease at the mild cognitive impairment stage. *Brain* **138**, 1327-1338.

[3] Skoldunger A, Johnell K, Winblad B, Wimo A (2013) Mortality and treatment costs have a great impact on the cost-effectiveness of disease modifying treatment in Alzheimer's disease--a simulation study. *Curr Alzheimer Res* **10**, 207-216.

[4] Skoldunger A, Wimo A, Johnell K (2012) Net costs of dementia in Sweden - An incidence based 10 year simulation study. *Int J Geriatr Psychiatry* **27**, 1112-1117.

[5] Zhang Y, Kivipelto M, Solomon A, Wimo A (2011) Cost-effectiveness of a health intervention program with risk reductions for getting demented: results of a Markov model in a Swedish/Finnish setting. *J Alzheimers Dis* **26**, 735-744.

[6] Burstrom K, Johannesson M, Diderichsen F (2001) Swedish population health-related quality of life results using the EQ-5D. *Qual Life Res* **10**, 621-635.

[7] Mesterton J, Wimo A, By A, Langworth S, Winblad B, Jonsson L (2010) Cross sectional observational study on the societal costs of Alzheimer's disease. *Curr Alzheimer Res* **7**, 358-367.

[8] Wimo A, Religa D, Spangberg K, Edlund AK, Winblad B, Eriksdotter M (2013) Costs of diagnosing dementia: results from SveDem, the Swedish Dementia Registry. *Int J Geriatr Psychiatry* **28**, 1039-1044.

[9] Handels R, Jönsson L, Garcia-Ptacek S, Eriksdotter M, Wimo A (2020) Controlling for selective drop-out in longitudinal dementia data: application to the SveDem registry *Alzheimer Dement*, doi: 10.1002/alz.12050.

[10] Skoldunger A, Fastbom J, Wimo A, Fratiglioni L, Johnell K (2015) Impact of inappropriate drug use on hospitalizations, mortality, and costs in older persons and persons with dementia: findings from the SNAC Study. *Drugs Aging* **32**, 671-678.
